# Analysis of retinal detachment resulted from post-operative endophthalmitis treated with 23G pars Plana Vitrectomy

**DOI:** 10.1186/s12886-021-02175-z

**Published:** 2021-12-01

**Authors:** Ying Zheng, Maria Casagrande, Spyridon Dimopoulos, Karl-Ulrich Bartz-Schmidt, Martin Stephan Spitzer, Christos Skevas

**Affiliations:** 1 Department of Ophthalmology, University Medical Center Hamburg-Eppendorf, Hamburg, Germany; 2 Department of Ophthalmology, Shanghai General Hospital Affiliated to Shanghai Jiaotong University, 100 Haining Road, Shanghai, 200080 China; 3 Department of Ophthalmology, Eberhard Karls University Medical Center, Tübingen, Germany

**Keywords:** Endophthalmitis, Retinal detachment, Pars Plana vitrectomy, 23 gauge

## Abstract

**Background:**

To evaluate the rate, risk factors, functional outcome and prognosis in eyes with retinal detachment after post-operative endophthalmitis treated with 23G Pars Plana Vitrectomy.

**Methods:**

Electronic patient files from 2009 until 2018 were screened for the presence of an endophthalmitis. Included were 116 eyes of 116 patients. This population was evaluated for the rate of retinal detachment after 23G Pars Plana Vitrectomy for endophthalmitis following cataract surgery or intravitreal injection. The main outcome measures were retinal detachment and visual acuity.

**Results:**

The reasons for endophthalmitis were previous cataract surgery in 78 patients and following intravitreal injection in 38 patients. The first clinical evidence of endophthalmitis was present in median 5 days after the triggering intervention. Twenty-five eyes (21.55%) developed a retinal detachment an average of 25 days after endophthalmitis. RD is significantly associated with preoperative visual acuity (*p* = 0.001).

**Conclusions:**

We emphasize the prognostic role of preoperative visual acuity in RD development of the endophthalmitis treated with 23G Pars Plana Vitrectomy.

## Background

Endophthalmitis (EO) is a severe intraocular inflammatory response. It is typically divided into exogenous, endogenous (systemic infection in an immune-compromised patient), or masquerade syndromes (large cell lymphoma). Exogenous is mostly postoperative (e.g., cataract surgery), but may also be post-traumatic or related to organisms with an ability to penetrate intact corneas. It can be classified as either culture-positive or culture-negative (sterile) [1, 2]. and further stratified into an acute form (within 6 weeks after surgery) which is the most common [3] and a delayed-onset form (more than 6 weeks after surgery). Reported incidence rates of post-operative endophthalmitis range differently due to different primary surgical interventions (e.g. post cataract surgery: 0.05–0.68%; post intravitreal injection: 0.02–0.03%). Acute endophthalmitis usually appears within one to 2 weeks after the primary surgical interventions (the median onset of post-operative endophthalmitis after cataract surgeries: 9 days; post intravitreal injection: 24 h) [4, 5].

Whatever form it may assume, endophthalmitis, which usually occurs with purulent inflammation of the intraocular fluids, such as the vitreous and the aqueous humor, is a serious and dangerous ocular condition, and can be very challenging for the vitreoretinal surgeon, because visibility can be severely compromised due to corneal edema, anterior chamber cells and non-transparent vitreous [6]. The toxins produced by the infecting pathogens and the resulting inflammatory responses can be destructive for the retina and lead to complications like retinal necrosis [6] or photoreceptor damage to the retina [7].

Retinal detachment (RD) is a complication of both endophthalmitis and the surgical procedures used in its treatment. The rate of RD in the management of endophthalmitis varies between 9 and 21% [4, 8–10]. RD was related to capsular rupture, noxious bacteria and an early additional procedure in the Endophthalmitis Vitrectomy Study (EVS). It led to a poor visual prognosis, with 27% of patients achieving a final best corrected visual acuity (BCVA) of 20/40 [10]. The EVS showed that early vitrectomy yielded better results when visual acuity had dropped to LP.

However, the approach to the treatment of endophthalmitis is not consistently agreed upon by vitreoretinal surgeons for all stages of visual acuity. The treatments involve Pars Plana Vitrectomy (PPV) or intravitreal broad-spectrum antibiotics with vitreous tap/biopsy (VTB).

The main objectives of this retrospective multi-center study were to evaluate the rate, risk factors, functional outcome and prognosis of RD after surgical treatment of patients with severe acute exogenous postoperative endophthalmitis having no BCVA exclusion criteria.

## Methods

In this retrospective study data of endophthalmitis patients from the departments of ophthalmology at the university clinic of Hamburg Eppendorf and the university clinic of the Eberhard Karls in Tübingen, Germany were evaluated.

Electronic patient files (Hamburg: IFA [ifa systems AG, Germany], Tübingen: Arzt-Informations- System [AIS]) were screened from 2009 until 2018 for the rate of endophthalmitis. Patients were diagnosed with endophthalmitis if they presented with characteristic endophthalmitis symptoms and signs (e.g., ocular pain, decreased vision, eyelid edema, conjunctival congestion, chemosis, anterior segment inflammation, hypopyon, vitritis, decreased red reflex, etc.) within 6 weeks following cataract surgery or intravitreal injection. In both centers the study included cases which were initially treated in those clinics or operated elsewhere and referred for treatment. Included were patients with endophthalmitis following cataract surgery and intravitreal injection who were treated with 23 G PPV and intravitreal medication (vancomycin 1 mg/0.1 mL and ceftazidime (2.225 mg/ 0.1 mL). Voriconazole has not been used with any patients during primary surgery. Patients with other reasons for endophthalmitis (endogenous source, post trauma, post filtrating surgery and post PPV) and patients treated with vitreous tap/biopsy were excluded. The criteria employed to diagnose endophthalmitis were fundoscopy, ultrasound with vitreous body infiltration, pain, hypopyon, anterior chamber inflammation and medical history. Recorded parameters were patient related data, pre-existing general health conditions, endophthalmitis-related data, BCVA and treatment. The population was further evaluated for the rate of RD after surgical treatment of endophthalmitis.

Vitrectomy did not include a peripheral shaving of the vitreous base and a posterior vitreous detachment (PVD) was not induced in any of the cases because posterior vitreous was already detached according to surgical reports. After completion of the vitrectomy a thorough but not too forceful examination of the peripheral retina was performed in order to locate any retinal breaks.

The study adhered to the tenets of the Declaration of Helsinki. The study was a retrospective data collection that was anonymized at the source. The study has been reviewed and approved by the ethics committee of Hamburg (PV7372) and written consent from the patients was not needed.

### Statistical analysis

All analyses were conducted using SPSS 19.0 software. Association was tested using the Chi-Square Test. Differences in time of factor variables were tested with the McNemar Test. The distribution of quantitative variables was given as median. Twenty-five (Q25%) and seventy-five (Q75%) quartiles were calculated. Statistical significance was set at *p* < 0.05.

## Results

This retrospective study included 116 eyes of 116 patients with endophthalmitis. The mean age was 74 years (range 48 to 96 years). Forty-seven patients (40.52%) were male and 69 patients (59.48%) were female. Out of these 116 patients 19% were treated for diabetes, 62.9% for arterial hypertension and 4.3% were immunosuppressed. Clinical evidence of endophthalmitis was reported in median 5 days after the causing incident (Q25: 3.00 days, Q75: 7.25 days). The median onset of endophthalmitis after cataract surgeries was 5 days (Q25: 3.00 days, Q75: 9.00 days) and post intravitreal injection was 4 days (Q25: 3 days, Q75: 5.00 days) (*p* = 0.019).

The reasons for endophthalmitis were previous cataract surgery in 78 patients and following intravitreal injection in 38 patients. Ninety-eight eyes were pseudophakic and 18 were phakic eyes.

Surgery was performed on the same day of presentation in both clinics. All patients were treated with PPV in combination with intravitreal antibiotics.

**Fig. 1:**
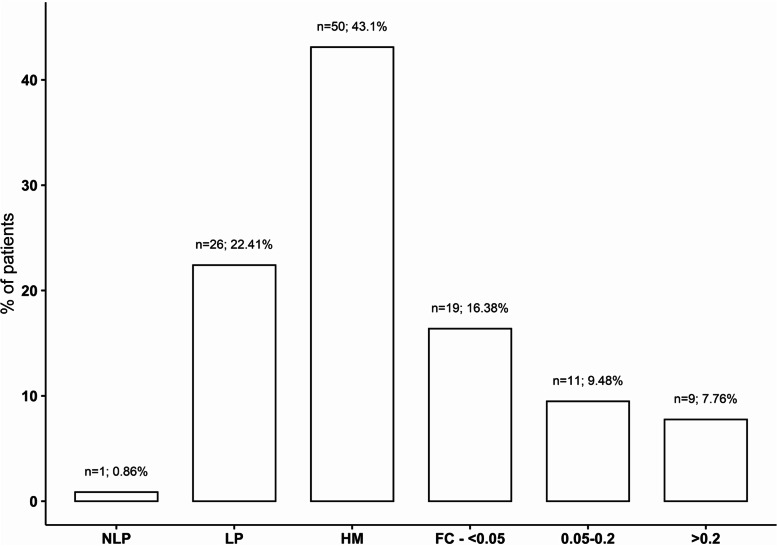
Distribution of preoperative visual acuity. The x-axis represents visual acuity and the y-axis the percentage of patients

Preoperative visual acuity was no light perception (NLP) in *n* = 1, light perception (LP) *n* = 26, hand movement (HM) *n* = 50, finger counting (FC) to < 0.05 *n* = 19, 0.05–0.2 *n* = 11 and > 0.2 *n* = 9 patients. The distribution of preoperative visual is displayed in Fig. 1.

An anterior chamber hypopyon was present in 81.9% of the patients.

RD occurred in 25 (21.55%) eyes with endophthalmitis after an average of 25.4 ± 16.8 days. RD was statistically significant associated with preoperative low visual acuity (*p* = 0.001). There is a slight tendency to lower incidence for eyes with better visual acuity (Spearman correlation rho = − 0.292, p = 0.001). For the distribution of RD by preoperative visual acuity see Table 1.

**Table 1:** The rate of RD by preoperative visual acuity

Visual acuity	RD
NLP	1:1 (100%)
LP	13:26 (50%)
HM	6:50 (12%)
FC- < 0.05	1:19 (5.3%)
0.05–0.2	2:11 (18.2%)
> 0.2	2:9 (22.2%)

Displays the rate of RD by preoperative visual acuity. Number of patients with RD: all patients with endophthalmitis (percentage) for different visual acuity groups.

**Fig. 2:**
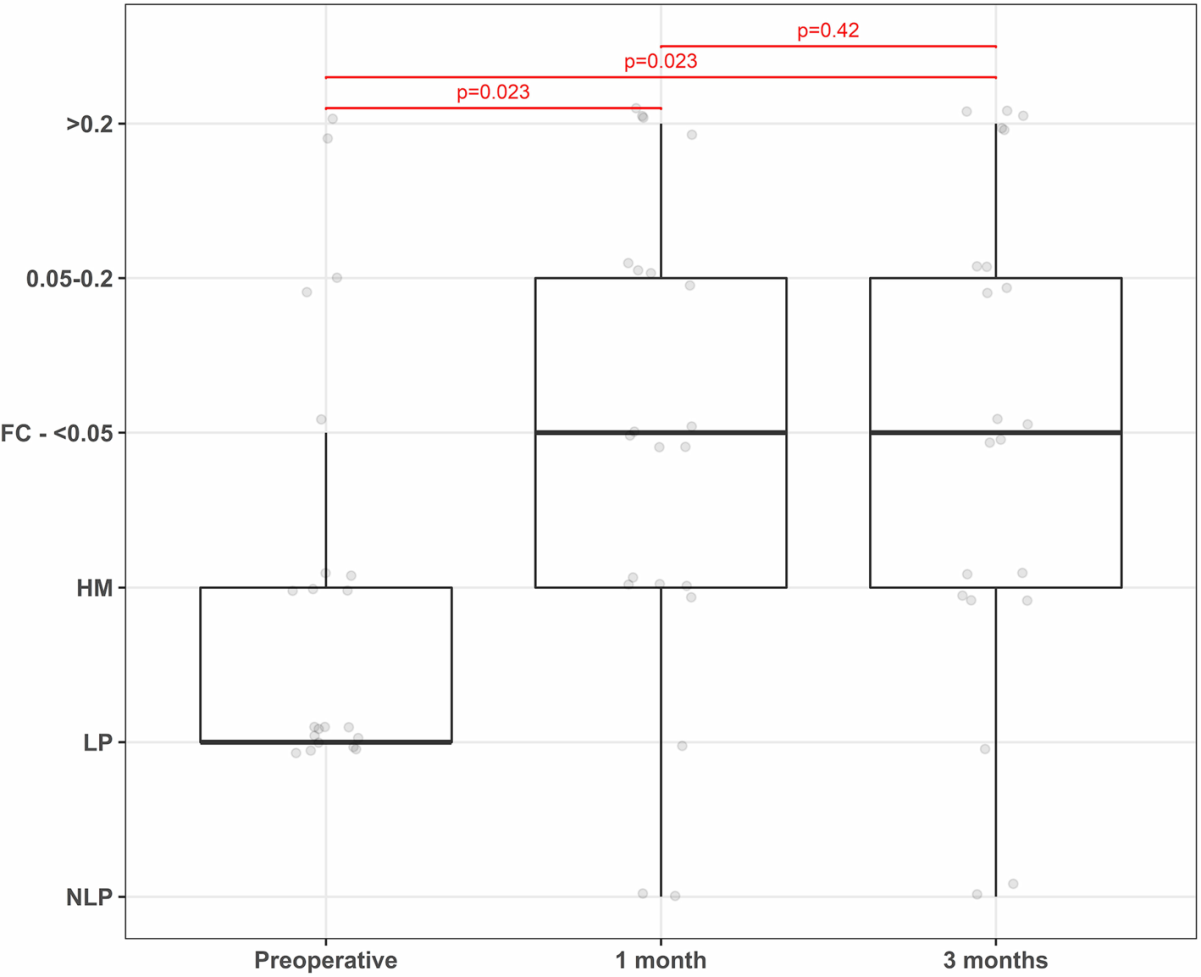
BCVA after the treatment

Following surgical intervention of the RD best corrected visual acuity (BCVA) improved significantly 1 month after the operation (*p* = 0.023) and stayed stable until the end of the follow-up period of 3 months after the operation (*p* = 0.42). The BCVA after the treatment is displayed in Fig. 2.

Six eyes (5.17%) were removed by enucleation due to phthisis bulbi. In these eyes no successful retinal reattachment was possible.

Ocular samples were obtained from vitreous sampling at the beginning of PPV. In 44.6% no growth was detected, in 50.7% gram + bacteria, in 3.1% gram – bacteria and in 1.6% fungal. No significant correlation was found between microbiological result and retinal detachment, *p* = 0.28.

There were no statistically significant differences between endophthalmitis following cataract surgery and intravitreal injection. See Table 2.

**Table 2:** Demographic data

	Causative procedures		*P*-value
	Post phaco	Post IVI	
**Eyes**	78	38	
**Patients**	78	38	
**Age in years (SD)**	73.49 (±9.87)	75.13 (±9.44)	0.388^1^
**Sex**			0.657^2^
Male (%)	30 (38.462)	17 (44.737)	
Female (%)	48 (61.538)	21 (55.263)	
**Diabetes**			0.514^2^
yes (%)	13 (16.667)	9 (23.684)	
no (%)	65 (83.333)	29 (76.316)	
**Arterial hypertension**			1.000^2^
yes (%)	49 (62.821)	24 (63.158)	
no (%)	29 (37.179)	14 (36.842)	
**Anterior chamber hypopyon**			0.405^2^
yes (%)	66 (84.615)	29 (76.316)	
no (%)	12 (15.385)	9 (23.684)	
**Retinal detachment**			0.416^2^
yes (%)	19 (24.359)	6 (15.789)	
no (%)	59 (75.641)	32 (84.211)	
**Enucleation (%)**	4 (5.128)	2 (5.263)	0.999^2^
**Preoperative visual acuity**			0.716^2^
NLP (%)	1 (1.282)	0 (0)	
LP (%)	18 (23.077)	8 (21.053)	
HM (%)	32 (41.026)	18 (47.368)	
FC - < 0.05 (%)	12 (15.385)	7 (18.421)	
0.05–0.2 (%)	7 (8.974)	4 (10.526)	
> 0.2 (%)	8 (10.256)	1 (2.632)	

Demographic data by reason for endophthalmitis.^1^-Independent T-Test,^2^-Chi-Square Test.

## Discussion

Infectious endophthalmitis is an inflammatory reaction that poses a high risk of severe visual loss. During any intraocular procedure, prevention of endophthalmitis should be a priority because of the multiple sources of contamination [11–13]. Povidone iodine (PI), a disinfectant and antiseptic agent, has been shown to be the only effective prophylactic method against postsurgical endophthalmitis [14]. The European Society of Cataract and Refractive Surgeons (ESCRS) and the American Academy of Ophthalmology (AAO) recommendations regarding PI use suggest 5% solution of PI before ocular surgery [15]. Alternative dosing strategies are being studied as well. For example, dilute PI was applied repetitively throughout cataract surgery (0.25% every 30 s), and 0.6% PI solution was also demonstrated to be an effective treatment in reducing conjunctival bacterial load and risk of needle contamination in patients undergoing intravitreal anti-vascular endothelial growth factor injection [15, 16].

A number of authors and studies addressed the problem of RD due to endophthalmitis and its surgical treatment. In the EVS, the rate of postoperative RD was 7.8% in the 20-gauge vitrectomy subgroup. In the future, due to the advancement of surgical techniques and technology, re-evaluation of this study’s results is needed [17, 18].

The evolution of the PPV technique with the introduction of 23-gauge and 25-gauge systems have made surgery less invasive. Nelsen et al. [8] reported RD rates of 21% after PPV treatment and 9% in eyes not treated with PPV, with an overall RD rate of 16% after surgical treatment of endophthalmitis. Olson and colleagues [8] reported an overall RD rate of 10% following post-surgical treatment, with a higher rate of 14% in post PPV eyes as well and Sridhar et al. reported a high RD rate of 21.4% in cases of acute endophthalmitis at the time of initial PPV or during follow up [19, 20]. Altan et al. reported that 13.8% of the subgroup treated with 20-gauge PPV led to a postoperative RD, while no RD developed in the subgroup treated with 25-gauge vitrectomy [21]. In a study by Almanjoumi et al., the rate was 10% after a 23-gauge PPV .

Endophthalmitis cases should be treated immediately after diagnosis in order to minimize retinal damage. In our study all cases were treated on the same day of presentation. Even after the inflammation has subsided, strict follow-up examinations are necessary due to onset of late RD. Cases of late RD up to a year after successful treatment of endophthalmitis have been reported. This could be caused by the production of various cytokines released into the vitreous cavity over a long period of time, due to the blood-ocular barrier breakdown. Retinal necrosis with tangential traction of retinal membranes can lead to formation of retinal breaks and rhegmatogenous RD. Tori et al. reported a rapid progression of proliferative vitreoretinopathy after endogenous bacterial endophthalmitis caused by meningitis [2, 22, 23]. The results of our study can verify this point of late onset RD and of necessary strict follow-ups.

The use of silicone oil has been reported by a number of authors in cases of intraoperative retinal breaks after post-surgical or following traumatic endophthalmitis [24–26]. Dave et al. reported high rates of RD at presentation and during follow up after initial surgery for endophthalmitis. All patients with RD were treated with silicone oil and the reattachment rates were deemed satisfactory [27]. Sridhar et al. reported a high RD rate of 21.4% in cases of acute endophthalmitis at the time of initial PPV or during follow up. Silicone oil proved to be effective in stabilizing the retina, but the BCVA was poor in almost all patients due to the severity of the cases [19]. Previously, due to the fear of infection behind the silicone oil bubble, there had been a reluctance to use silicone oil as a tamponade agent for endophthalmitis [28]. Later, silicone oil was proved to have an antibacterial and antifungal effect in vitro. The possible mechanisms of its antimicrobial activity that were reported are nutritional deprivation and toxicity [29]. The dosage of intravitreal antibiotics in eyes treated with silicone oil injection still remains controversial. Hegazy’s study demonstrated a retinal toxicity in silicone oil-filled rabbit eyes, when the full dose of intravitreal antibiotics was used [30]. However, those results might not apply to the human eyes. Still we believe it is wise to reduce the dose of intravitreal drugs to about 25% of the dose that is usually injected because all intravitreal drugs will only distribute in the small aqueous phase surrounding the silicone bubble.

On the microbiological side, the results of the organisms identified in our study are in accordance with other studies and there was no statistically significant correlation between the microbiological findings and the occurrence of RD or the initial BCVA (no statistical significance between Gram+ bacteria and severity of endophthalmitis, BCVA and rate of RD) [31, 32].

The treatment strategy of a severe endophthalmitis is complicated. Time is of essence, and the goal is to eiminate the infection and administer antibiotics. The intravitreal injection of antibiotics and the vitrectomy are the standard and main therapeutic options. Every option has advantages and disadvantages. While vitrectomy allows for the infection to be removed as complete as possible, the procedure is often not possible, since vitreoretinal surgeons and vitreoretinal operating rooms are relatively scarce. The vitreous tap/biopsy and intravitreal antibiotics injection have their own advantage. For example, they offer a smaller sample and permit earlier intravitreal antibiotics injections and microbiology tests [33]. Vitrectomy has evolved over the years after the EVS study (smaller gauges, faster surgical procedures, minimally invasive) but the rates of RD vary and can still be high in severe cases of endophthalmitis as demonstrated not only in the EVS study but by other authors using modern PPV techniques (23 and 25 gauge systems) [19, 21, 22, 28].

The high rate of RD in our cohort cannot be attributed to iatrogenic intraoperative breaks or to the vitreous sampling. No shaving of the vitreous base was performed and the posterior hyaloid was not forcfully detached because it was already detached in all cases. We also did not use undiluted vitreous for vitreous sampling but rather diluted. Chiquet et al. have reported that undiluted vitreous sampling at the start of PPV leads to hypotony, with a potential risk of vitreoretinal tractions, haemorrhages and RD. This can be avoided using diluted samples, since both samples have the same microbiological efficiency using PCR [34].

In our study, retinal detachment is statistically significant associated with preoperative visual acuity, which is similar to the findings of Doft et al. stating that RD is more likely to develop in patients who have the most severe presentation with visual acuity of LP only [9]. On the other hand, Chiquet et al. reported that other risk factors for RD in patients who had a vitrectomy after cataract surgery were diabetes and vasculitis [20]. Our study could not find a statistical significant correlation between microbiological findings (especially Gram+ bacteria), diabetes mellitus, immunosuppression and RD rate, but the increased signs of severity of endophthalmitis were more visible in patients with lower initial BCVA. Vitrectomy offers the advantage of an as-complete-as-possible evacuation of the infection but is associated with a spectrum of complications like RD.

Some of the limitations of most of the studies in the literature today dealing with this very complex problem are the retrospective nature, lack of a defined treatment protocol, treatment by multiple vitreoretinal surgeons and exclusion of cases due to the complexity of the disease and poor visual prognosis of this condition. These facts also apply to our study, but the large number of cases via the inclusion of two retinal centers makes us optimistic that our conclusions could shed some light on this complex issue.

## Conclusions

The findings of this study suggest that modern 23G vitrectomy technique seems not to lower the rate of RD after vitrectomy for endophthalmitis. The risk of retinal detachment still remains high in spite of the updated vitreoretinal techniques, especially with a higher cutting rate. And we also emphasize the prognostic role of preoperative visual acuity in RD development of the endophthalmitis treated with 23G Pars Plana Vitrectomy, presumably due to the inflammatory effect on the vitreous and retina.

## Data Availability

The datasets used and/or analyzed during the current study are available from the corresponding author on reasonable request.

## Abbreviations

AAO: The American Academy of Ophthalmology
AIS: Arzt-Informations- System
BCVA: Best corrected visual acuity
EO: Endophthalmitis
EVS: Endophthalmitis Vitrectomy Study
ESCRS: The European Society of Cataract and Refractive Surgeons
FC: Finger counting
HM: Hand movement
LP: Light perception
NLP: No light perception
PI: Povidone iodine
PPV: Pars plana vitrectomy
PVD: Posterior vitreous detachment
RD: Retinal detachment
VTB: Vitreous tap/biopsy
