# Correction to: Analysis of retinal detachment resulted from post-operative endophthalmitis treated with 23G pars Plana Vitrectomy

**DOI:** 10.1186/s12886-022-02258-5

**Published:** 2022-02-08

**Authors:** Ying Zheng, Maria Casagrande, Spyridon Dimopoulos, Karl-Ulrich Bartz-Schmidt, Martin Stephan Spitzer, Christos Skevas

**Affiliations:** 1 Department of Ophthalmology, University Medical Center Hamburg-Eppen- dorf, Hamburg, Germany; 2 Department of Ophthalmology, Shanghai General Hospital Afliated to Shanghai Jiaotong University, 100 Haining Road, Shang- hai, 200080 China; 3 Department of Ophthalmology, Eberhard Karls University Medical Center, Tübingen, Germany

**Correction to: BMC Ophthalmol 21, 414 (2021)**

**https://doi.org/10.1186/s12886-021-02175-z**

Following the publication of the original article [1], we were notified that the author tagging was incorrect.

Originally published names: Zheng Ying, Casagrande Maria, Dimopoulos Spyridon, Bartz Schmidt Karl Ulrich, Spitzer Matin Stephan and Skevas Christos.

Corrected names: Ying Zheng, Maria Casagrande, Spyridon Dimopoulos, Karl Ulrich Bartz Schmidt, Martin Stephan Spitzer and Christos Skevas.

Also, Spitzer Martin Stephan was originally spelled as Spitzer Matin Stephan.

The original article has been corrected.
